# Comparison of Chip Inlet Geometry in Microfluidic Devices for Cell Studies

**DOI:** 10.3390/molecules21060778

**Published:** 2016-06-15

**Authors:** Yung-Shin Sun

**Affiliations:** Department of Physics, Fu-Jen Catholic University, New Taipei City 24205, Taiwan; 089957@mail.fju.edu.tw; Tel.: +886-2-2905-2585

**Keywords:** microfluidic chip, inlet geometry, cell separation, cell capture, fluidic shear stress

## Abstract

Micro-fabricated devices integrated with fluidic components provide an *in vitro* platform for cell studies best mimicking the *in vivo* micro-environment. These devices are capable of creating precise and controllable surroundings of pH value, temperature, salt concentration, and other physical or chemical stimuli. Various cell studies such as chemotaxis and electrotaxis can be performed by using such devices. Moreover, microfluidic chips are designed and fabricated for applications in cell separations such as circulating tumor cell (CTC) chips. Usually, there are two most commonly used inlets in connecting the microfluidic chip to sample/reagent loading tubes: the vertical (top-loading) inlet and the parallel (in-line) inlet. Designing this macro-to-micro interface is believed to play an important role in device performance. In this study, by using the commercial COMSOL Multiphysics software, we compared the cell capture behavior in microfluidic devices with different inlet types and sample flow velocities. Three different inlets were constructed: the vertical inlet, the parallel inlet, and the vertically parallel inlet. We investigated the velocity field, the flow streamline, the cell capture rate, and the laminar shear stress in these inlets. It was concluded that the inlet should be designed depending on the experimental purpose, *i.e.*, one wants to maximize or minimize cell capture. Also, although increasing the flow velocity could reduce cell sedimentation, too high shear stresses are thought harmful to cells. Our findings indicate that the inlet design and flow velocity are crucial and should be well considered in fabricating microfluidic devices for cell studies.

## 1. Introduction

*In vivo* cells work properly by responding to their environmental chemical and physical stimuli such as chemical gradients of various growth factors and mechanical interactions with the extracellular matrix (ECM). Traditionally, Petri dishes and microplates are commonly used for *in vitro* cell studies because of their easy operation in cell culture and observation. However, in using such macro-scaled devices, the consumption of reagents and cells is great, and also cells grow in a static (non-circulating) environment. To overcome these hurdles, micro-fabricated devices integrated with fluidic components have recently become popular as an alternative platform for cell studies in a more controllable manner. These microfluidic chips are capable of creating a precise micro-environment of chemical and physical stimuli while minimizing the consumption of cells and reagents and maintaining cells in circulating surrounding. They can be made of glass substrates, silicon wafers, polymethylmethacrylate (PMMA) substrates, polyethyleneterephthalate (PET) substrates, or polydimethylsiloxane (PDMS) polymers [1,2,3]. PMMA is a transparent thermoplastic which is cheap and easy to process using laser ablation. PDMS is a transparent, biocompatible polymer which is permeable to gas, making it suitable for long-term cell culture and observation. Since developed, microfluidic devices have been applied to cell studies under a stable micro-environment of controllable chemical and physical stimuli. For example, microfluidic chips were used to study how cells respond to certain chemicals, a phenomenon termed chemotaxis [1,4,5], and to electric fields (EFs), a phenomenon named electrotaxis [6,7,8,9].

Recently, microfluidic devices have been commonly and widely used in cell separations because of their high throughput, high precision, automation, and miniaturization. One example is the circulating tumor cell (CTC) chip which enables the isolation of rare tumor cells in bloodstreams of cancer patients (~1–100 CTCs per 10^9^ blood cells). These chips can be classified into two types: separations based on physical properties such as sizes, shapes, and charges, and separations based on chemical properties such as surface markers and active chemical groups [10,11,12,13,14]. Hou *et al.* reported using a spiral microchannel with inherent centrifugal forces for continuous, size-based separation of CTCs from blood [15]. This microfluidic chip was optimized to reach a recovery rate of >85% and a high throughput of 3 μL/h Lee *et al.* fabricated a contraction-expansion array (CEA) microchannel device to, based on inertial lift force and Dean flow, separate cancer cells from whole blood at low Reynolds number (Re) [16]. A recovery rate of 99.1%, a blood cell rejection ratio of 88.9%, and a throughput of 1.1 × 10^8^ cells/min were achieved. Zhao *et al.* developed a platform to capture and isolate cells using a 3D DNA network composed of repeated adhesive aptamer domains extending over tens of micrometers into the solution [17]. It was demonstrated that the 3D DNA network significantly enhanced the capture efficiency of lymphoblast CCRF-CEM cells over monovalent aptamers and antibodies, yet maintained a high purity of the captured cells. Another example is microfluidic-based separation and isolation of bacteria from blood [18,19,20,21]. Lee *et al.* developed a magnetic microfluidic device for clearing bacteria and endotoxin from the bloodstream. This device was used to remove *Escherichia coli*, a Gram-negative bacterium, from bovine whole blood at a volume flow rate of 60 μL/h, resulting in almost 100% clearance [21]. Wu *et al.* showed using a microfluidic chip to, based on soft inertial force-induced migration, separate bacteria from human blood cells. This device, with an active size of 3 mm^2^, was demonstrated to successfully separate *Escherichia coli* from human red blood cells at high cell concentrations (above 10^8^/μL) and a sample volume flow rate of up to 18 μL/min. 

In a microfluidic cell separation chip, the macro-to-micro interface, connecting macroscopic tubes and microscope fluidic channels, plays an important role in device performance. An ideal interface features smooth connection, automated operation, and zero dead volume [22,23]. For typical cell sizes of 10–20 μm, a microfluidic device with a width and a height of less than 100 μm could cause cell sedimentation near the interface [24]. This phenomenon occurs near the chip inlet most likely when the flow rate is too low, and such clogging could further result in flow irregularities and channel blockage. Usually, there are two ways to connect loading tubes to microfluidic channels: the vertical inlet scheme and the parallel inlet scheme. In a vertical inlet chip, the loading tube is perpendicularly connected to the fluidic channel. This approach is more commonly used because of its easy fabrication and compatibility with multilayered geometry [13,25]. In a parallel inlet chip, the tube is inserted in parallel into the fluidic channel. In addition, the sample flow velocity is a crucial parameter in performing cell separation experiments. As the velocity is too low, cell sedimentation near the inlet could cause channel blockage. A higher velocity overcomes the problem of clogging as well as increases the throughput, but it could do damage to cells due to an excessive shear stress. As reported, fluidic shear stresses of as low as 0.25–0.6 Pa could interface cell attachment, and even higher values of stresses (0.5–10 Pa) could remove adherent cells [26]. Moreover, 0.8 to 1.5 Pa of laminar shear stresses could induce cell alignment in the direction of flow [27], and values of 0.1–1 Pa were shown to affect cellular morphology and permeability [26]. A shear stress of 60 Pa could lyse around 25% of the population in leukocytes [28], and shear stresses of 2–120 Pa significantly brought down the number in smooth muscle cells [29]. Also, using a microfluidic chip, Lo *et al.* reported that the production of reactive oxygen species (ROS) in lung cancer cells increased in response to an increasing shear stress from 0.0048 Pa to 0.0192 Pa [30]. 

In this paper, we compared cell sedimentation in microfluidic devices with different inlet geometries and sample flow velocities. Using the commercial COMSOL Multiphysics software, three different macro-to-micro interfaces were constructed for studying cell capture within fluidic channels: the vertical inlet, the parallel inlet, and the vertically parallel inlet. We studied the velocity field, the flow streamline, the laminar shear stress, and the cell capture rate in these inlets. We concluded that the inlet should be designed depending on the experimental purpose: in cell separations based on chemical bindings, one wants to capture as many cells as possible, and in cell separations based on physical properties, one wants to minimize cell attachment onto the wall. In addition, although increasing the flow velocity could reduce cell sedimentation, too high shear stresses are considered harmful to cells under investigation. These results provide scientists and engineers with useful guidelines in designing and fabricating microfluidic devices for cell studies. 

## 2. Results and Discussion

### 2.1. Velocity Field and Flow Streamline

**Figure 1:**
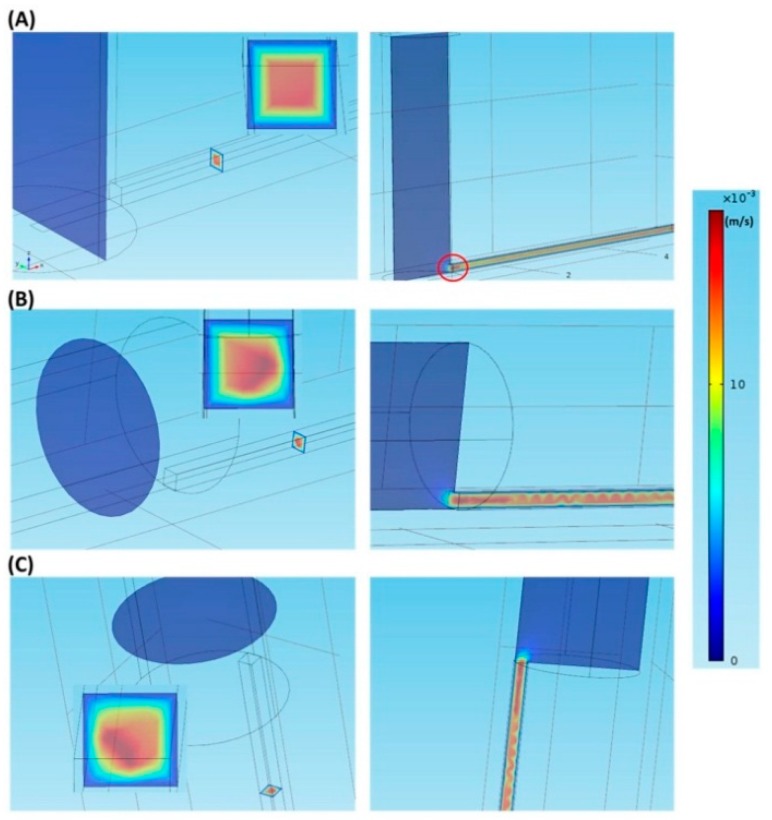
The velocity fields in different inlets at a flow velocity of 0.0001 m/s. (**A**) The vertical inlet; (**B**) The parallel inlet; (**C**) The vertically parallel inlet.

Figure 1A shows the velocity field in the vertical inlet at a flow velocity of 0.0001 m/s. As shown in the left panel, a square laminar flow profile appears in the cross-section perpendicular to the flow direction. In the right panel, where the cross-section is parallel to the flow direction, the flow velocity is maximal near the macro-to-micro interface (see the red circle). The velocity field profiles are similar under other flow velocities. The velocity field, at a flow velocity of 0.0001 m/s, in the parallel inlet is shown in Figure 1B. The left panel indicates that the laminar flow profile resembles a quadrant in the cross-section perpendicular to the flow direction. In the right panel, where the cross-section is parallel to the flow direction, a wavelike velocity field appears around 300 μm after passing the interface. A similar velocity field at the same flow velocity is observed in the vertically parallel inlet, as shown in Figure 1C. Again, in both the parallel and the vertically parallel inlets, the velocity field profiles are similar under other flow velocities. 

**Figure 2:**
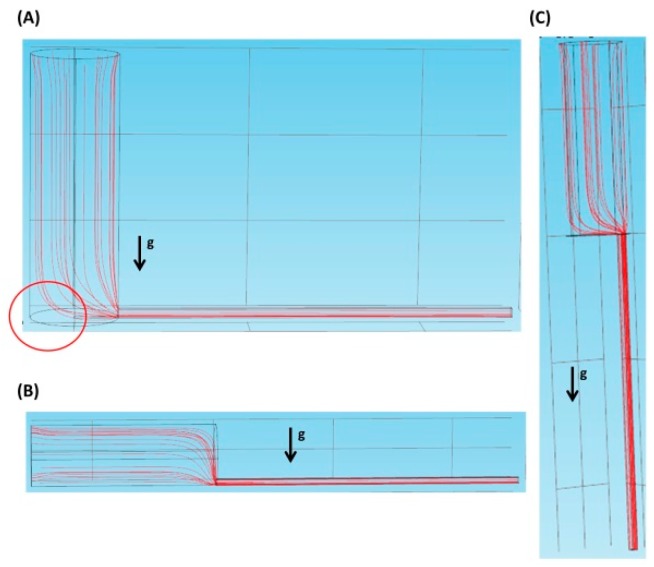
The flow streamlines in different inlets at a flow velocity of 0.0001 m/s. (**A**) The vertical inlet; (**B**) The parallel inlet; (**C**) The vertically parallel inlet.

Figure 2A–C show the flow streamlines of the vertical inlet, the parallel inlet, and the vertically parallel inlet, respectively, at a flow velocity of 0.0001 m/s. In Figure 2A, under the effect of gravity, there could be a zero-velocity zone (see the red circle) where cells remain static once they are there. This might in turn result in cell sedimentation. As shown in Figure 2B, gravity could help cells move down and pass through the interface along the flow streamline. In Figure 2C, the direction of gravity and that of flow are the same, so cells could easily enter the microscopic channel with the aid of gravity. Therefore, in the latter two cases, we speculate that very little sedimentation is observed.

### 2.2. Cell Sedimentation

**Figure 3:**
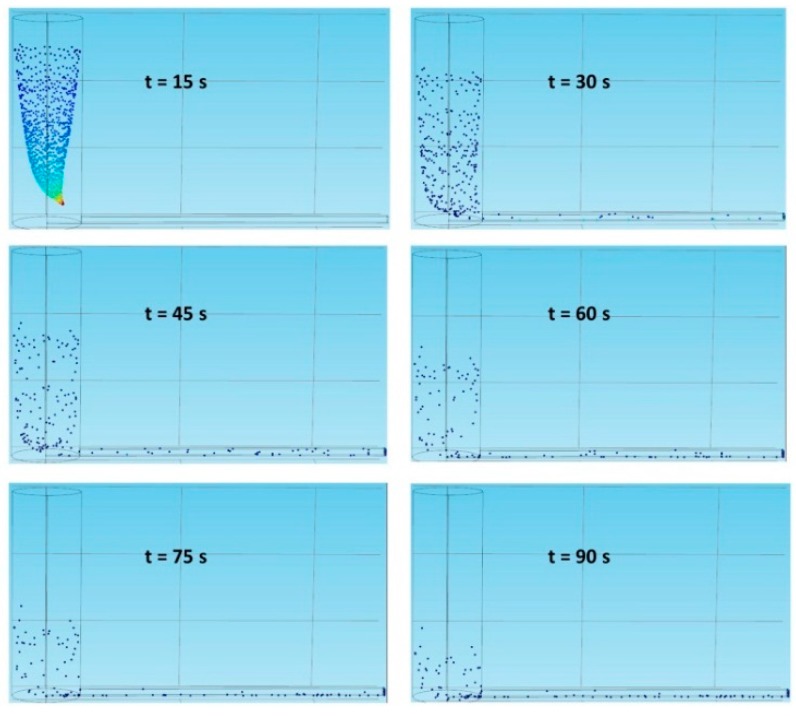
The particle trajectories in the vertical inlet under the Freeze wall condition at a flow velocity of 0.0001 m/s.

To evaluate how inlet geometry and flow velocity affect cell sedimentation, we inject 1000 cells from the inlet, wait for a suitable time period, and calculate how many cells are collected in the micro-channel outlet. Cell sedimentation refers to cells being captured by the walls of the tubes and the channels. Table 1 lists different flow velocities used in the simulation. Figure 3 shows the particle trajectories in the vertical inlet under the Freeze wall condition at a flow velocity of 0.0001 m/s. At time points of 0, 15, 30, 45, 60, 75, 90, 105, 120, 135, and 150 s, the cell numbers counted are 0, 700, 827, 879, 890, 893, 895, 895, 895, 895, and 895, respectively. Since these numbers reach a plateau after 75 s, the number 890 is selected as the saturation value in the vertical inlet at a flow velocity of 0.0001 m/s. A similar way is used to find the saturated cell number in the outlet under a specified condition. 

**Figure 4:**
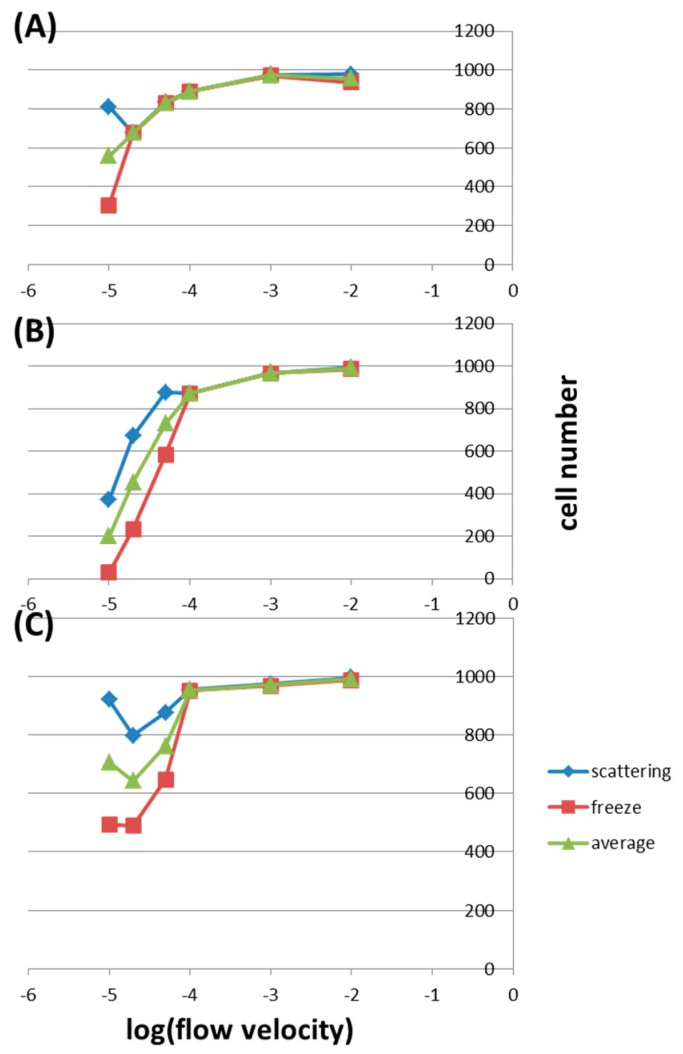
The cell numbers counted in the outlet under different wall conditions and flow velocities in (**A**) the vertical inlet; (**B**) the parallel inlet; and (**C**) the vertically parallel inlet.

Figure 4A shows the cell numbers in the vertical inlet under different wall conditions and flow velocities. In this inlet, the cell numbers are almost the same using both diffuse scattering and freeze conditions except at a very low flow velocity of 0.00001 m/s. In general, the average cell number decreases with decreasing flow velocity. For example, the average cell numbers are 974.5, 890, and 558.5 at flow velocities of 0.001, 0.0001, and 0.00001 m/s, respectively. Figure 4B shows the cell numbers in the parallel inlet under different conditions. Here, at flow velocities lower than 0.0001 m/s, the cell numbers are higher in the diffuse scattering condition than those in the freeze condition. Also, after this velocity, there is a sharp decrease in cell numbers with decreasing flow velocity. For example, the average cell number decreases from 990.5 to 872 when the velocity is lowered from 0.01 to 0.0001 m/s, but the number drops from 872 to 199.5 when the velocity is further decreased to 0.00001 m/s. This may be because cells sediment to the bottom of the tube before entering the micro-channel as a result of gravity. As shown in Figure 4C, in the vertically parallel inlet, the average cell numbers are almost the same for flow velocities higher than 0.0001 m/s, being 991, 971, and 953 for 0.01, 0.001, and 0.0001 m/s, respectively. 

**Figure 5:**
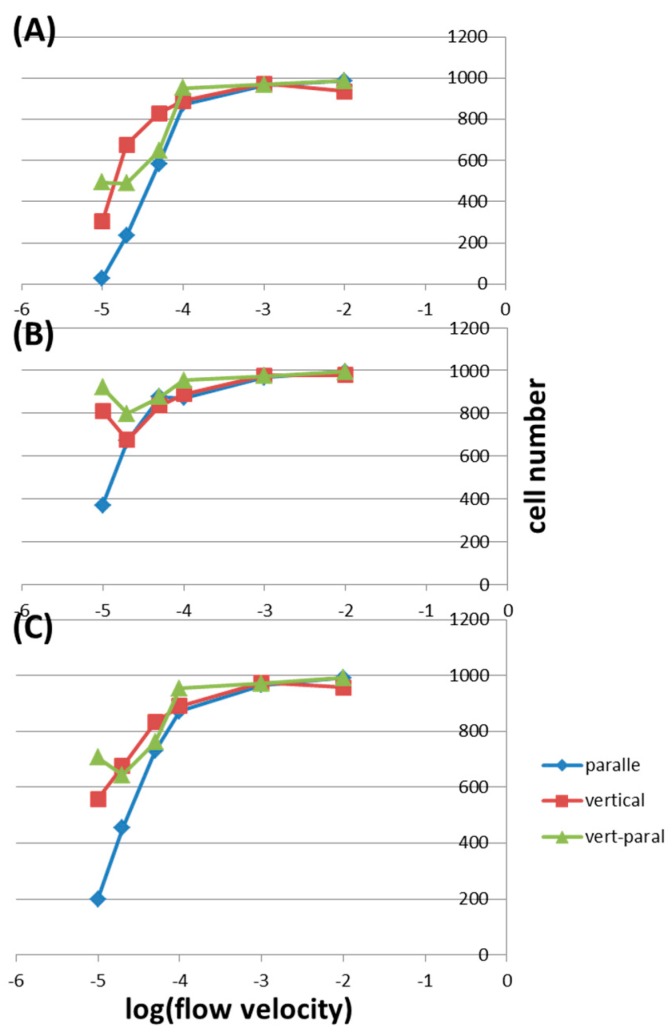
The cell numbers counted in the outlet under different inlets and flow velocities by using (**A**) the Freeze wall condition and (**B**) the Diffuse scattering wall condition; (**C**) The average cell numbers of (**A**,**B**).

After this point, the average cell numbers decrease obviously with deceasing velocity down to 0.00002 m/s, being 761.5 and 742.5 for 0.001 and 0.0001 m/s, respectively. However, this number increases to 706.5 at 0.00001 m/s. We speculate that (1) for velocities between 0.01 and 0.0001 m/s, the force due to flow is strong enough to bring almost all cells across the interface; (2) for velocities between 0.0001 and 0.00002 m/s, the gravity forces some cells to sediment onto the interface of the tube end; (3) at a velocity of 0.00001 m/s, cells about to sediment as described in (2) have more time to move along the streamline and across the interface without being greatly affected by the gravity. Depending on the application of the microfluidic chip, the inner wall of the fluidic channel should be modified correspondingly. To capture as many cells as possible within the micro-channel, the wall is modified to be hydrophilic or coated with specific biomolecules (e.g., antibodies or antigens). In this case, the freeze wall condition is used. And to minimize cell attachment onto the wall, it is modified to be hydrophobic, where the diffuse scattering wall condition is used. Therefore, it is of interest to discuss cell capture under different wall conditions. Figure 5A shows the cell numbers in the freeze wall condition under different inlets and flow velocities. For velocities higher than 0.0001 m/s, the cell numbers remain almost the same in all three inlets and all different velocities. With lower velocities, the cell number decreases with decreasing velocity and with geometry being changed from vertical to vertically parallel and to parallel inlets. For example, at a flow velocity of 0.00005 m/s, the cell numbers are 829, 647, and 584 in the vertical inlet, the vertically parallel inlet, and the parallel inlet, respectively. When the velocity is further lowered to 0.00002 m/s, these values decrease to 676, 488, and 235 correspondingly. At an even lower velocity of 0.00001 m/s, only 29 cells reach the end of the micro-channel in the parallel inlet. In the parallel inlet, the gravity overcomes the flow force at low velocities, causing cell sedimentation to the bottom of the tube and the channel. Figure 5B shows the cell numbers in the Diffuse scattering wall condition. For velocities higher than 0.00005 m/s, the cell numbers don’t change much in all three inlets. At a velocity of 0.00002 m/s, this number is the highest in the vertically parallel inlet, being 797, and is slightly lower in other inlets, being 676 and 671 in the vertical one and the parallel one, respectively. In the parallel inlet under this wall condition, cells could be scattered off the wall few times before finally captured by the surface, resulting in much more cells collected in the outlet compared to what are observed in the Freeze wall condition.

Figure 5C shows the average cell numbers under different inlets and flow velocities. This is the case when the inner wall of the fluidic channel is not specially modified: cells are subject to the freeze wall condition and the diffuse scattering wall condition with equal probabilities. The cell numbers are almost the same in all three inlets for velocities higher than 0.0001 m/s, and with lower velocities, the cell number in the vertical inlet is the highest and that in parallel inlet is the lowest. For example, at a flow velocity of 0.00002 m/s, the average cell numbers are 676, 642.5, and 453 in the vertical inlet, the vertically parallel inlet, and the parallel inlet, respectively. These results have to do with the determining effect of gravity in the parallel inlet under low flow velocities.

### 2.3. Effects of Shear Stress

Fluidic shear stresses are known to influence cells in various aspects including attachment, morphology, alignment, living cycle, and damage. As described in the Introduction, shear stresses of 0.25–0.6 Pa could affect cell attachment, those of 0.5–10 Pa could remove adherent cells, those of 0.1–1.5 Pa could change cell morphology and alignment, and those of a few tens of Pa could cause interface cell propagation. As listed in Table 1, flow velocities of 0.001 and 0.01 m/s produce fluidic shear stresses of 7.54 and 75.4 Pa, respectively, considered high enough to damage cells. Flow velocities of 0.0001 and 0.00005 m/s, corresponding to shear stresses of 0.754 and 0.377 Pa, respectively, could possibly affect cell morphology, alignment, and attachment. Only flow velocities lower than 0.00002 m/s are thought to make no effect to cells. However, these too low velocities greatly reduce the throughput in microfluidic applications. For example, using the current chip, analyzing a sample of 100 μL takes 0.2, 2, 20, and 200 min in flow velocities of 0.01, 0.001, 0.0001, and 0.00001 m/s, respectively. Therefore, a compromise between the throughput and the effect of shear stress leads to optimal flow velocities of around 0.00005 m/s. Referring back to Figure 5, under such velocities, one should apply the parallel inlet for capturing as many cells as possible (Figure 5A, the freeze wall condition) and use the vertically parallel inlet for minimizing cell attachment (Figure 5B, the diffuse scattering wall condition). Effects of flow velocity and the resulting fluidic shear stress should be addressed in designing and fabricating microfluidic devices of different dimensions.

## 3. Materials and Methods 

### 3.1. Different Inlet Geometries

The COMSOL Multiphysics commercial software (version 4.4, COMSOL Inc., Burlington, MA, USA) package is used for constructing different macro-to-micro interfaces. In these geometries, a macroscopic tube with a radius of 500 μm and a length of 3 mm is connected to a microscopic channel with a width of 100 μm, a depth of 100 μm, and a length of 5 mm. 

**Figure 6:**
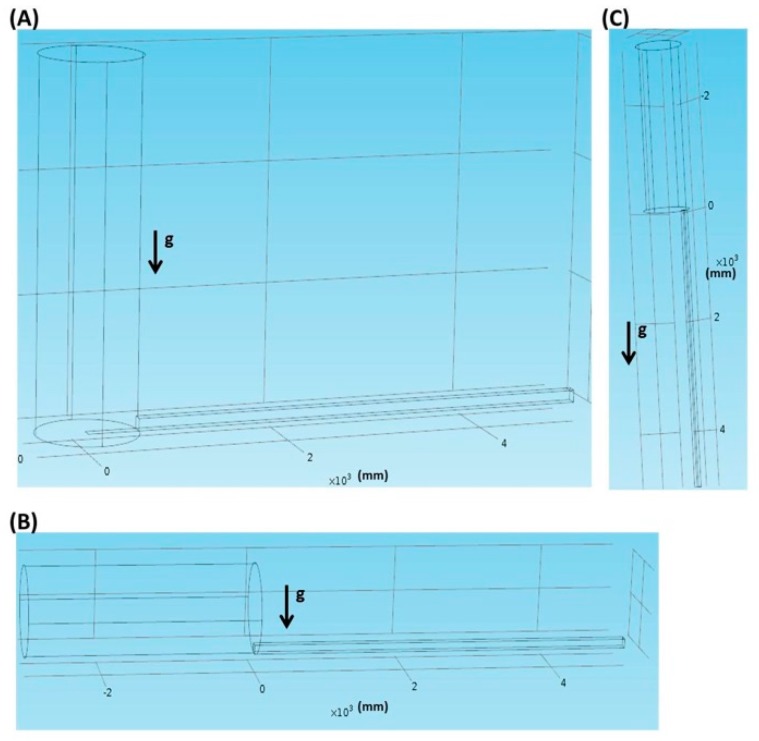
Different inlet geometries. (**A**) The vertical inlet; (**B**) The parallel inlet; (**C**) The vertically parallel inlet.

As shown in Figure 6A, in the vertical inlet, the tube is perpendicularly connected, along the direction of gravity, to the fluidic channel. In Figure 6B, in the parallel inlet, the tube is inserted in parallel into the fluidic channel. In the vertically parallel inlet as shown in Figure 6C, the tube is inserted in parallel, along the direction of gravity, into the fluidic channel.

### 3.2. COMSOL Simulation

To simulate the velocity field within the microfluidic channel, the “Laminar Flow” module is used with the liquid set to be water. Liquid flow velocities of 0.00001, 0.00002, 0.00005, 0.0001, 0.001, and 0.01 m/s, corresponding to volume flow rates of 0.471, 0.942, 2.355, 4.71, 47.1, and 471 μL/min, respectively, are set at the inlet of the loading tube. Since the cross-sectional area of the tube is 0.785 mm^2^ and that of the micro-channel area is only 0.01 mm^2^, the volume flow rates are equal in both the tube and the channel, but the liquid flow velocities within the channel are 78.5 times higher than those within the tube. The laminar shear stress (τ) within the fluidic channel is related to the volume flow rate (*Q*), the fluidic viscosity (η), and the dimension of the channel (height *h* and width *w*) as [30,31]:

(1) $$\tau =12\frac{Q\eta }{h^{2}w}$$

It depends on the flow rate and the channel dimension but not on the inlet type. By setting η = 0.0008 Pa·s for water and *w* = *h* = 100 μm, the shear stresses are calculated to be 0.07536, 0.15072, 0.3768, 0.7536, 7.536, and 75.36 Pa under different volume flow rates. 

For particle tracking within the microfluidic channel, the “Particle Tracking for Fluid Flow” module is used. A particle density of 1050 kg/m^3^ and a particle diameter of 10 μm are set to mimic cells. And since cells can either be captured by or scattered off channel walls, two different wall conditions are set: Freeze and Diffuse scattering. In the Freeze condition, the final velocity of a particle (***v***) equals to its initial velocity (***v_i_***) when striking the wall. In the Diffuse scattering condition, the following equations hold, where θ and φ are the scattering angles:

(2) $$v_{t1}=\left(v_{i}\cdot v_{i}-v_{n}^{2}\right)sin\phi ,\;v_{t2}=\left(v_{i}\cdot v_{i}-v_{n}^{2}\right)cos\phi$$

and:

(3) $$v_{n}=\left|v_{i}\right|cos\theta$$

Particles are subject to two forces: the gravity force can be expressed as:

(4) $$F_{g}=m_{p}g\frac{\left(\rho_{p}-\rho \right)}{\rho_{p}}$$

where *m_p_*, ρ*_p_*, and ρ are the mass of the particle, the density of the particle, and the density of the fluid, respectively; the Stokes drag force can be expressed as:

(5) $$F_{d}=\frac{18\mu }{\rho_{p}d_{p}^{2}}m_{p}\left(u-v\right)$$

where *μ*, *d_p_*, *u*, and *v* are the viscosity of the fluid, the diameter of the particle, the flow velocity, and the particle velocity, respectively. A finite element mesh is created, containing 25,593 tetrahedral elements, 9030 prism elements, 4850 triangular elements, 498 edge elements, 186 pyramid elements, 72 quadrilateral elements, and 18 vertex elements. For the “Laminar Flow” module, a stationary study is conducted. In the “Particle Tracking for Fluid Flow” module, a time-dependent study is conducted with the timing steps depending on the flow velocity.

## 4. Conclusions

We have investigated the effects of inlet geometries and flow velocities on cell capturing within microfluidic devices. Regardless of the inlet, higher flow velocities effectively reduce cell attachment onto the inner wall of the sample loading tube and the microfluidic channel. However, since the fluidic shear stress is directly proportional to the flow velocity, too high velocities could affect cells in various aspects including attachment, morphology, alignment, living cycle, and damage. From the simulation results, to attain a balance between increasing the throughput and reducing the effect of shear stress, optimal flow velocities of around 0.00005 m/s are attained. Under such velocities, the parallel inlet should be applied if one wants to capture as many cells as possible, and the vertically parallel inlet should be used if one wants to minimize cell capturing. This work provides useful guidelines for those designing and fabricating microfluidic devices for cell studies.

## Figures and Tables

**Table 1:** Different flow velocities and corresponding mass flow rates used in the simulation. Calculated shear stresses are also listed.

Flow Velocity ^1^ (m/s)	0.00001	0.00002	0.00005	0.0001	0.001	0.01
Mass flow rate (μL/min)	0.471	0.942	2.355	4.71	47.1	471
Shear stress (Pa)	0.07536	0.15072	0.3768	0.7536	7.536	75.36

^1^ Flow velocities are set at the inlet of the loading tube.
